# Native Wolbachia infection dynamics across Aedes albopictus (Diptera: Culicidae) populations in Hawai‘i

**DOI:** 10.21203/rs.3.rs-9715732/v1

**Published:** 2026-06-10

**Authors:** Sangwoo Seok, Leena Salama, Adam E. Vorsino, Mark K. H. Leong, William P. Haines, Christopher M. Jacobsen, Omar S. Akbari, Eric P. Caragata, Yoosook Lee

**Affiliations:** University of Florida; University of Florida; Pacific Islands Fish & Wildlife Office; Environmental Health, Tripler Army Medical Center; Department of Land and Natural Resources; Hawaii Department of Health; University of California; University of Florida; University of Florida

**Keywords:** Wolbachia, mosquito, Aedes albopictus, Incompatible insect technique, microbe-microbe dynamics

## Abstract

**Background:**

*Aedes albopictus*, an important vector of arboviruses such as dengue, chikungunya, and Zika, is a widespread invasive mosquito species in the Pacific Islands Countries and Territories, including Hawai‘i. *Wolbachia*, an endosymbiotic bacterium, widely used for mosquito control through its cytoplasmic incompatibility and pathogen blocking properties. Two *Wolbachia* strains, *w*AlbA and *w*AlbB, naturally inhabit *Aedes albopictus* unlike *Aedes aegypti*. However, their infection patterns in Hawai‘i remain poorly characterized and may influence the success of future *Wolbachia*-based control efforts.

**Methods:**

Using quantitative PCR, we characterized the *Wolbachia* infection status of *Ae. albopictus* collected from two Hawaiian Islands: O‘ahu and the Island of Hawai‘i. We assessed the influence of sex, island, temperature, and precipitation on the prevalence and density of *w*AlbA and *w*AlbB, as well as interactions between the two strains.

**Results:**

Overall, 98.5% (*N* = 399) of individuals were infected with *Wolbachia*. All infected specimens carried *w*AlbB, but only 28.6% were superinfected with *w*AlbA. Infection dynamics were strongly sex-specific, with females significantly more likely to harbor *w*AlbA, and *Wolbachia* density was also significantly higher in females. The prevalence of *w*AlbA was associated with temperature and precipitation at the collection site. However, *w*AlbB prevalence varied with the island and precipitation. In superinfected individuals, *w*AlbB consistently exhibited higher density than *w*AlbA, while *w*AlbB density was higher in superinfected individuals than in those with *w*AlbB mono-infection. Interestingly, both strains showed higher densities in females than in males. Strain densities were positively correlated in females but not in males, indicating sex-specific interactions.

**Conclusions:**

By characterizing the *Wolbachia* infection dynamics of *Ae. albopictus* populations in Hawai‘i, these findings establish an important baseline for biocontrol efforts. We demonstrate that *Ae. albopictus* mosquitoes in Hawai‘i were either superinfected or carry only *w*AlbB, with lower-than-expected *w*AlbA infection, which may complicate the design and effectiveness of *Wolbachia*-based control strategies. Our results highlight that future population suppression projects involving *Wolbachia* and cytoplasmic incompatibility in Hawai’i must take into account varying patterns of *Wolbachia* infection in the region prior to biocontrol strategy development and implementation.

## Background

Between 2011 and 2020, the number of outbreaks caused by arboviruses, viruses transmitted by arthropods such as mosquitoes, in the Pacific Islands Countries and Territories (PICT) more than doubled compared to the previous decade [1]. A major cause of these outbreaks was *Aedes albopictus* (Skuse, 1894), commonly known as the Asian tiger mosquito, which is known to spread key arboviruses such as dengue, chikungunya, Zika, Ross River, yellow fever, and Japanese encephalitis in the PICT region [2]. This mosquito has a widespread distribution throughout the Pacific where it is found in 16 territories [1]. It arrived in the Hawaiian Islands prior to the turn of the twentieth century, and quickly displaced *Aedes aegypti*, another invasive vector species [3], which now has a comparatively limited distribution [4]. The displacement of *Ae. aegypti* by *Ae. albopictus* has been documented across multiple regions worldwide [5, 6].

*Aedes albopictus* naturally harbors *Wolbachia pipientis*, an intracellular, maternally inherited bacterial endosymbiont that is common amongst insects [7–10]. This is different from *Ae. aegypti*, which does not have any naturally occurring *Wolbachia* in natural populations [11]. *Wolbachia* is currently described as a single species but can be classified into phylogenetically distinct lineages known as supergroups, of which, supergroups A and B are known to infect mosquitoes and other dipterans [7, 12]. Populations of *Ae. albopictus* are commonly superinfected, meaning that they typically harbor two distinct strains of *Wolbachia* called *w*AlbA and *w*AlbB, which belong to supergroups A and B, respectively [8, 13].

In the past two decades, *Wolbachia* has gained popularity as a versatile tool for replacing [14] or suppressing [15] mosquito populations, with this utility arising due to the symbiont’s manipulations of mosquito biology. These interventions rely on the transinfection of mosquitoes, where *Wolbachia* is transferred from a donor species to a target species via embryonic microinjection [16, 17]. Common donor species include naturally infected mosquitoes such as *Ae. albopictus* or *Culex pipiens* or fruit flies like *Drosophila melanogaster*. *Wolbachia*-mediated population suppression, also known as the Incompatible Insect Technique (IIT), exploits a reproductive manipulation caused by *Wolbachia* called Cytoplasmic Incompatibility (CI), which occurs when *Wolbachia*-infected males mate with -uninfected females. Due to CI, those females lay inviable eggs that fail to hatch. The IIT involves mass releases of male *Wolbachia*-infected mosquitoes, which seek out and mate with wild *Wolbachia-*free females. On a sufficiently large scale, these releases can reduce the size of a target mosquito population by significantly reducing the production of viable progeny. Large-scale IIT programs have been implemented for the control of *Ae. aegypti* or *Ae. albopictus* in several countries including China, Mexico, and Singapore and achieved 65–91% population reduction during the trial period [15, 18, 19]. However, the success of these approaches depends critically on the underlying *Wolbachia* infection structure of target populations, which can vary spatially, temporally, and between sexes.

Mass release programs relying on the IIT have regularly demonstrated successful reduction in the size of the target mosquito population. In California, a 95% reduction of wild *Ae. aegypti* population was observed after *Wolbachia*-infected males were released over 26 weeks [20]. Similarly, the technique was successfully used to suppress populations of *Ae. aegypti* and *Aedes polynesiensis* on private atolls in French Polynesia [21]. One study in northern Australia, observed 80% suppression of an *Ae. aegypti* population after *Wolbachia*-infected males were released over a 20-week period [22]. Meanwhile, a long-term study in Singapore encompassing more than 700,000 residents has seen mosquito abundance in the treated areas drop by about 85% and an accompanying drop in dengue cases amongst residents in those areas [19]. Despite these successes, variation in *Wolbachia* infection structure across target populations can fundamentally alter compatibility outcomes and reduce suppression efficiency.

A major concern with the IIT and other mosquito control strategies that rely on population suppression is that they leave a vacant ecological niche that can be re-filled by the same or another mosquito species, necessitating frequent retreatment and increased biosecurity. Therefore, these techniques are often considered for controlling invasive mosquito species such as *Ae. aegypti* and *Ae. albopictus* in non-native ranges and continued monitoring is essential. The potential for the introduction of a new invasive mosquito species into Hawai‘i is exhibited by the previous detections of *Anopheles punctipennis* and *Culex tarsalis* in Honolulu [23]. Nonetheless, the northern Australia study also demonstrated a 97% suppression rate in one of the treatment sites, eleven months after releases ended, suggesting that not all vacant niches become filled immediately after the intervention ceases [22].

*Wolbachia* transinfections can also cause pathogen blocking, the restriction of infection, replication, and transmission of key pathogens in the tissues of *Wolbachia*-infected mosquitoes [24]. Pathogen blocking and CI form the basis of population replacement strategies, which seek to immunize mosquito populations against pathogens of medical importance [25, 26]. Field applications using population replacement have been conducted for several *Ae. aegypti* populations transinfected with the *w*Mel *Wolbachia* strain in Latin America, Asia, and five locations in the Pacific [27]. An independent program has successfully established a population of *Ae. aegypti* transinfected with the *w*AlbB strain in Malaysia and reported decreased dengue incidence in the release area [28].

The dynamics of *Wolbachia* infection in *Ae. albopictus* populations represent a key constraint on the design and effectiveness of *Wolbachia*-based interventions. *Wolbachia* infection rates and superinfection rates can vary greatly within and between populations of *Ae. albopictus* [29–31], and the rate of superinfection is typically higher in females than in males [30]. Additionally, the two native *Wolbachia* strains cause minor and inconsistent pathogen blocking and are unsuitable to prevent arbovirus transmission on their own [33, 34]. Finally, the presence of native infections can complicate suppression due to bidirectional incompatibility, which occurs when CI occurs between mosquitoes infected by different, incompatible *Wolbachia* strains [34]. Without bidirectional incompatibility, viable eggs are produced by mating of released males and wild females, making population suppression impossible.

Consequently, IIT or replacement strategies targeting *Ae. albopictus* may produce incompatibility by clearing the native *Wolbachia* with antibiotics and then using a novel transinfection to cause CI with the two native strains. Alternatively, they may overlay a third strain in superinfection with *w*AlbA and *w*AlbB, as long as the triple infection is incompatible with the two native strains [36]. For example, the release trial of triple-infected *Ae. albopictus* in Guangzhou, China, demonstrated up to a 65% reduction in mosquito population compared to control sites [15].

Accordingly, there is a need to understand the baseline dynamics of *Wolbachia* infection in an *Ae. albopictus* population prior to the implementation of genetic biocontrol tools, such as the IIT, in order to assess their feasibility. In Hawai‘i, data on the *Wolbachia* infection status of *Ae. albopictus* remains limited with a single study examining a small number of female specimens from the Island of Hawai‘i (N = 9) [8]. To that end, we used quantitative PCR-based screening to perform a rigorous characterization of the prevalence of the two native *Wolbachia* strains in *Ae. albopictus* populations of O‘ahu and the Island of Hawai‘i, as these islands are likely to be priority targets for future *Wolbachia*-based mosquito control due to high human population density and a history of dengue outbreaks [3, 37].

## Methods

### Sampling

Adult *Ae. albopictus* were collected across O‘ahu and the Island of Hawai‘i between April 2023 and July 2024 using BG-sentinel traps, sweeping nets, or ovitraps. Sampling sites were selected to focus on areas with high human population density that are likely to be priority targets for future *Wolbachia*-based mosquito control on both islands. All sites were located within 1 km of residential and commercial areas. Accordingly, sampling sites on O‘ahu included Aliamanu Military Reservation (AMR), Fort Shafter (FS), Mānoa (MA), Miliani Mauka (MM), Helemano (HE), Schofield Barracks (SB), Tripler Army Medical Center (TAMC), and Wheeler Army Airfield (WA). On the Island of Hawai‘i, samples were collected from Hōlualoa (HO), Hilo (HI), Kawaihae (KA), and Miloli‘i (MI). Mosquitoes were collected together with *Ae. aegypti*, *Ae. vexans*, *Culex quinquefasciatus, Wyeomyia mitchellii*. *Aedes albopictus* was identified based on its characteristic dorsal stripe pattern, and sex was determined by examination of the antennae. A total of 405 samples were collected, including 31 males and 39 females from the Island of Hawai‘i, and 154 males and 181 females from O‘ahu. GPS coordinates and a breakdown of specimen count by sex are provided in Table 1.

### DNA extractions

The whole body of each specimen was placed into a tube containing 180 μL of squash buffer, which was prepared by dissolving 0.6057 g of Tris Base, 0.1861 g of EDTA, and 1.4619 g of NaCl in distilled water to a final volume of 500 mL, along with a single bead. Then, the samples were homogenized using TissueLyser II (Qiagen, Germany) at 19.5 Hz for three minutes. Following the homogenization, 1 μL of proteinase K was added to each tube. The mixtures were incubated at 56°C for five minutes and then boiled at 100°C for five minutes. The extracted DNA was subsequently cooled on ice for 5 minutes and stored at −20°C before use.

#### Detection of Wolbachia infection

To detect the presence of *Wolbachia* and quantify *Wolbachia* titer in each specimen, three primer sets were employed in quantitative PCR (qPCR) assay. *Wolbachia* strain-specific primers were used to identify infection of *w*AlbA (*w*AlbA_F: 5’-GTAGTATTTACCCCAGCAG-3’; *w*AlbA_R: 5’-ATCTGCACCAGTAGTTTCG-3) [36], and *w*AlbB (*w*AlbB_F: 5’-CCTTACCTCCTGCACAACAA-3’; *w*AlbB_R: 5’-GGATTGTCCAGTGGCCTTA-3’) [38]. The third set of primers targeting the mosquito hemothorax gene (qHTH-F: 5’-TGGTCCTATATTGGCGAGCTA-3’; qHTH-R: 5’-TCGTTTTTGCAAGAAGGTCA-3’) [36] served as a host control gene and facilitated relative quantification of each strain. Each qPCR reaction contained 1μL of each 5 μM primer, 5 μL of PowerUp^™^ SYBR^™^ Green Master Mix (Applied Biosystems, USA), 1 μL of molecular grade water, and 2 μL of extracted DNA, which was diluted 1:5 in molecular water prior to use. Reactions were performed using a CFX96 Touch Real-Time PCR Detection System (Bio-Rad, USA). The qPCR run profile was 95°C for 3 min, then 40 cycles of 95°C for 15 s and 60°C for 30 s, followed by a melt-curve analysis (65–95°C with 0.5°C increments, for 5s per increment).

For each specimen, independent, duplicate qPCR reactions were performed for each gene in order to quantify the presence and abundance of *w*AlbA and *w*AlbB. Relative titers of each strain from each specimen were estimated as mean normalized expression values were calculated using Q-Gene [39]. To assess the accuracy of these measurements, the relative standard error value was calculated as well. An error threshold was set at 20%, and qPCR was repeated for each specimen were repeated if that threshold was exceeded.

### Climatic data

Data for two climatic variables (annual mean temperature and annual precipitation) corresponding to the location of specimen collections were obtained from the WorldClim database version 2.1 [40]. These data corresponded to the average climatic conditions at our sampling sites between 1970 and 2000, and had a spatial resolution of 30 arc seconds. Though absolute temperatures and precipitation metrics have likely shifted since the development of the 1970–2000 WorldClim dataset, these historical averages were used under the assumption that the relative climatic gradients between the distinct collection sites remain consistent. Therefore, these variables serve as a proxy for the persisting micro-climatic differences driving localized *Wolbachia* dynamics.

### Statistical analysis

All statistical analyses were performed using R version 4.5.0 [40]. To ensure sufficient statistical power, sampling sites where less than five specimens of either sex were collected (sites HE, MA, WA, and HI) were excluded from analyses.

To examine variation in the prevalence of *Wolbachia* strains, we fitted generalized linear models (GLMs) for *w*AlbA and *w*AlbB data with binomial error distributions, using mosquito sex and island as categorical predictors and standardized annual mean temperature and annual precipitation as continuous predictors. To assess differences in superinfection rates, Fisher’s exact tests were performed comparing the proportion of individuals with *w*AlbB mono-infection and superinfection (*w*AlbA and *w*AlbB) between sexes and between islands.

Comparison of *Wolbachia* density was performed using non-parametric Mann-Whitney U tests after density data were determined to have a nonparametric distribution. Comparison of *w*AlbA and *w*AlbB densities was performed for all superinfected individuals, while *w*AlbB density was compared between superinfected individuals and individuals with *w*AlbB mono-infection. Additionally, relative density data were transformed using the Box-Cox method to meet the assumption of normality. Linear models were then used to examine the effects of sex, island, temperature, and precipitation on the relative densities of each strain. Pearson’s correlation analyses were conducted to assess the relationship between the relative densities of the two strains for superinfected individuals, followed by additional analyses comparing female and male specimens. Inter-island comparisons were conducted to assess regional differences in infection dynamics. While the chosen statistical methods are well-suited for unequal groups, the disparity in sample sizes between O‘ahu (N = 335) and the Island of Hawai‘i (N = 70) inherently reduces statistical power for generalized linear modeling of the latter cohort. Consequently, while the mathematical comparisons are valid, island-specific findings should be interpreted with this constraint in mind. However, the collection sites were not included as a predictor variable in any of these models due to the disproportionate specimen numbers collected between sites.

## Results

### Wolbachia infection prevalence

**Figure 1:**
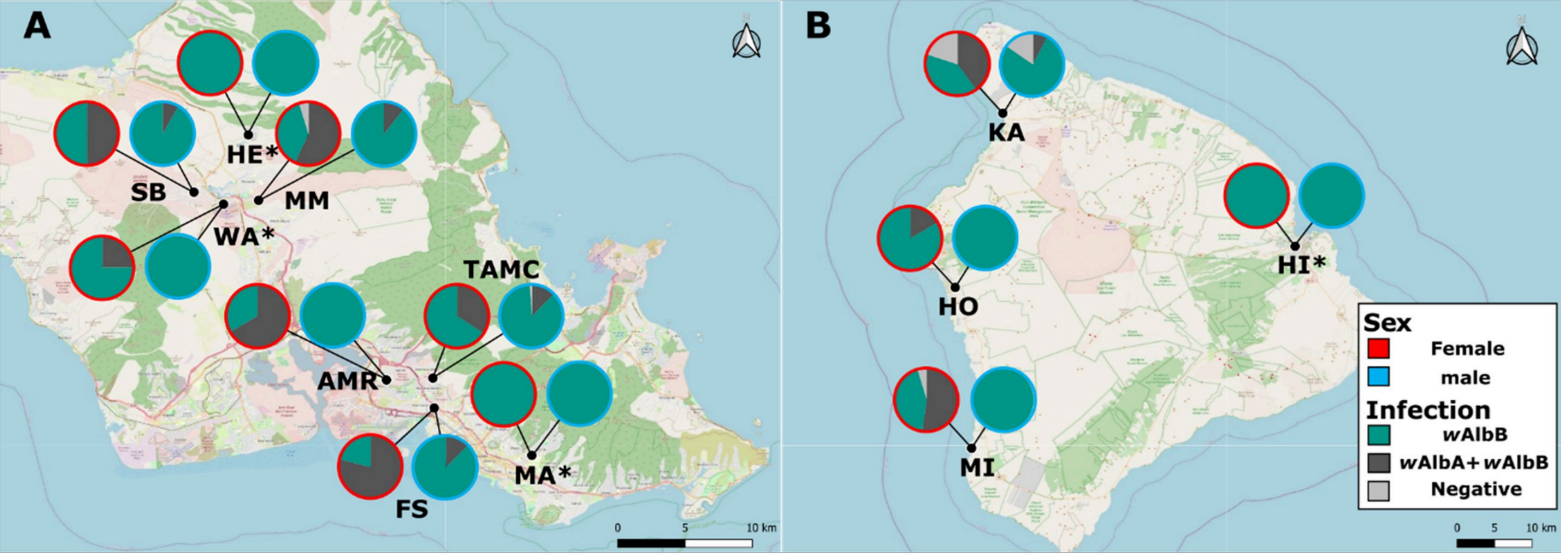
Distribution of *Wolbachia* infection status in *Aedes albopictus* in (A) O‘ahu and (B) Island of Hawai‘i: superinfected (dark gray), *w*AlbB only (green), and uninfected (light gray). Red and blue outlines indicate female and male, respectively. Sampling sites on O‘ahu: AMR = Aliamanu Military Reservation, FS = Fort Shafter, MA = Mānoa, MM = Mililani Mauka, HE = Helemano, SB = Schofield Barracks, TAMC = Tripler Army Medical Center, and WA = Wheeler Army Airfield. Sampling sites on the Island of Hawai‘i: HO = Hōlualoa, HI = Hilo, KA = Kawaihae, MI = Milolii. Populations marked with an asterisk were not included in statistical analyses due to small sample sizes.

A total of 399 out of 405 *Ae. albopictus* (98.5%) across the two Hawaiian Islands were found to be infected with *Wolbachia* (Table 1, Fig. 1). All 399 infected specimens were positive for *w*AlbB, and a total of 116 (28.6%) individuals were superinfected, harboring both the *w*AlbA and *w*AlbB strains. No individuals harbored *w*AlbA without also carrying *w*AlbB. Infection patterns were similar between islands. For female specimens collected from O‘ahu, 0.6% were uninfected, 53.0% were infected with *w*AlbB only, and 46.4% were superinfected. For males from O‘ahu island, 0.6% were uninfected, 89.0% had a *w*AlbB mono-infection, and the final 10.4% were superinfected. For female specimens from the Island of Hawai‘i, 5.1% were uninfected, 56.4% had a *w*AlbB mono-infection, and 38.5% were superinfected. For males from the Island of Hawai‘i, 6.5% were uninfected, 90.3% were *w*AlbB only, and 3.2% were superinfected. Subsequent statistical analyses utilized the data from 377 specimens, excluding 22 individuals from four sites due to low collection numbers (< 5 specimens collected from either sex).

### Climatic conditions

Climatic conditions varied across the eight sampling sites that were included in statistical analyses. On O‘ahu, the annual mean temperature ranged from 22.7 to 24.9 °C, while the annual precipitation ranged from 900 to 1593 mm across sites. On the Island of Hawai‘i, the annual mean temperature ranged from 21.8 to 24.6 °C, and the annual precipitation ranged from 920 to 1803 mm. The lowest temperature was recorded at HO, Island of Hawai‘i (21.8 °C), whereas the highest reached at FS, O‘ahu (24.9 °C). The lowest precipitation was recorded at AMR, O‘ahu (900 mm), whereas the highest precipitation reached at HO, Island of Hawai‘i (1803 mm).

#### Prevalence of Wolbachia

Evaluation of *w*AlbA prevalence using a binomial GLM revealed that sex was a significant predictor, with males being 88.8% less likely to be infected with *w*AlbA strain than females (binomial GLM, odds ratio = 0.112, *z* = −7.339, *P* < 0.001). There was no significant difference in *w*AlbA prevalence due to the island where specimens were collected (binomial GLM, odds ratio = 0.527, *z* = −1.793, *P* = 0.073). However, *w*AlbA prevalence was significantly higher at sites where the annual mean temperature was higher (binomial GLM, odds ratio = 1.353, *z* = 2.332, *P* = 0.020) and the annual precipitation was higher (binomial GLM, odds ratio = 1.364, *z* = 2.426, *P* = 0.015).

The prevalence of *w*AlbB, again using a binomial GLM, exhibited contrasting patterns to those seen for *w*AlbA. Sex was not a significant predictor of *w*AlbB prevalence (binomial GLM, odds ratio = 1.659, z = 0.550, *P* = 0.583). However, island was a significant factor, with *w*AlbB prevalence being significantly lower on the Island of Hawai‘i than on O‘ahu (binomial GLM, odds ratio = 0.021, z = −2.769, *P* = 0.006). For the climatic variables, the annual precipitation was positively associated with *w*AlbB prevalence (binomial GLM, odds ratio = 10.955, z = 2.059, *P* = 0.040), but temperature did not have a significant influence (binomial GLM, odds ratio = 3.545, z = 1.239, *P* = 0.215).

**Figure 2:**
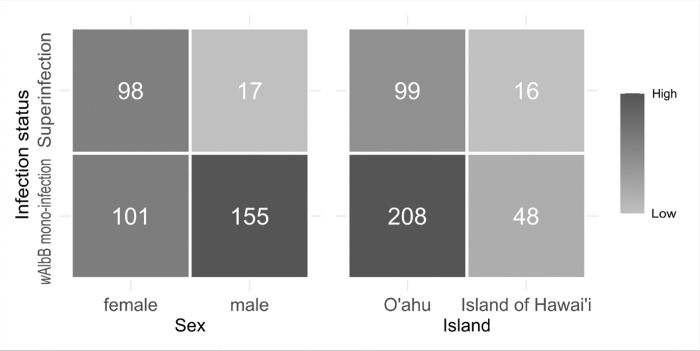
Contingency table heatmap displaying the distribution of wAlbB mono-infection and superinfection (*w*AlbA + *w*AlbB) status by sex (left) and island (right) from eight sampling sites across Hawaiian Islands. Color intensity reflects the number of individuals in each infection status.

Of the individuals infected with *w*AlbB (n = 371), 31.0% (n = 115) were superinfected with both *w*AlbA and *w*AlbB. Fisher’s exact tests revealed a significant difference in superinfection rates between sexes (odds ratio = 8.793, *P* < 0.001), indicating approximately 8.8-fold higher odds of superinfection in females than in males, but no significant difference in superinfection rate was observed between islands (odds ratio = 0.701, *P* = 0.299) (Fig. 2).

#### Relative density of Wolbachia

**Figure 3:**
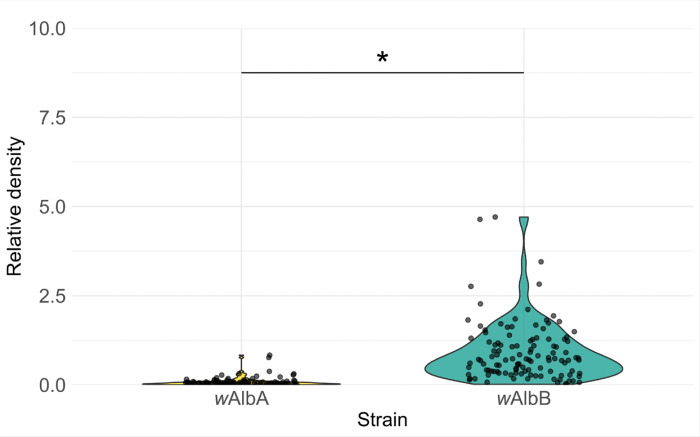
Comparison between the relative densities of *w*AlbA and *w*AlbB in superinfected individuals. The relative densities were calculated using qHTH as the reference gene. The asterisk marks indicate a statistically significant difference between two groups.

**Figure 4:**
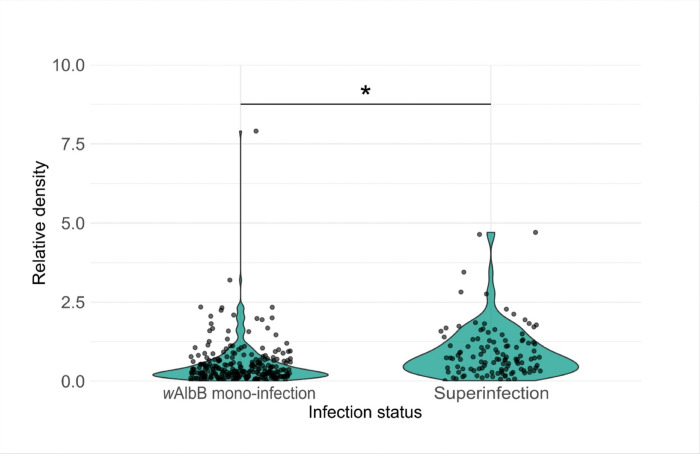
Comparison between the relative densities of individuals with *w*AlbB mono-infection and superinfection (*w*AlbA and *w*AlbB). The relative densities were calculated using qHTH as the reference gene. The asterisk marks indicate a statistically significant difference between two groups.

The relative density of *w*AlbA and *w*AlbB was compared between specimens. Among superinfected individuals, *w*AlbB exhibited significantly higher relative densities than *w*AlbA (Mann-Whitney U test, U = 557, *P* < 0.001), with a 15.21-fold higher median density (Fig. 3). We also observed that median *w*AlbB density was 2.22-fold higher amongst superinfected individuals compared to those with *w*AlbB mono-infection (Mann-Whitney U test, U = 8792.5, *P* < 0.001) (Fig. 4).

**Figure 5:**
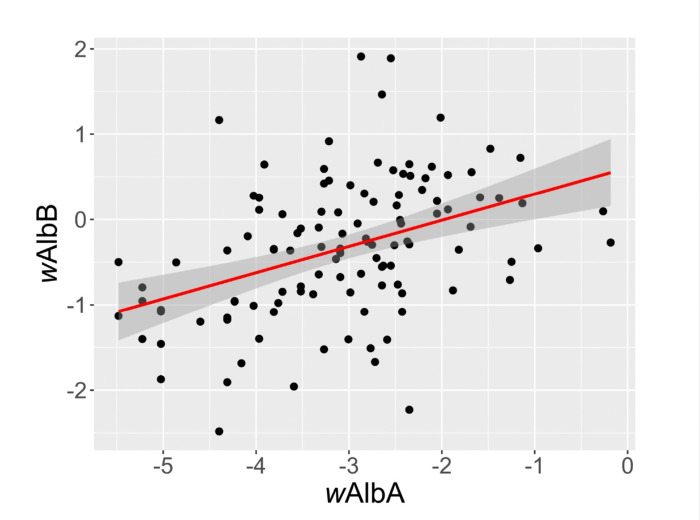
Correlation between relative densities of *w*AlbA and *w*AlbB in superinfected individuals (r = 0.410, *P* < 0.001). Relative densities were transformed using Box-Cox methods to meet the normality of density data before the correlation analysis.

**Figure 6:**
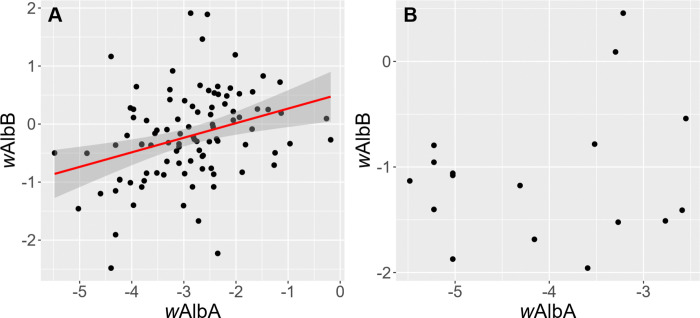
Correlation between relative densities of *w*AlbA and *w*AlbB in superinfected (A) females (r = 0.324, *P* = 0.001) and (B) males (r = 0.202, *P* = 0.437). Relative densities were transformed using Box-Cox methods to meet the normality of density data before the correlation analysis.

Linear models revealed significant effects of sex on the relative density of both *w*AlbA and *w*AlbB among superinfected individuals. For both *w*AlbA (linear model, t = −24.392, *P* < 0.001) and *w*AlbB (linear model, t = −7.556, *P* < 0.001), males exhibited significantly lower density than females. For *w*AlbA, there was no influence of island, temperature or precipitation on the relative density. However, for *w*AlbB, precipitation was a significant predictor (linear model, t = 3.263, *P* = 0.001). Densities of the two strains in superinfected mosquitoes of both sexes displayed a moderately positive but significant correlation (Fig. 5, r = 0.410, *P* < 0.001). Splitting those data by sex revealed a significant, but weaker correlation for females (*r* = 0.324, *P* = 0.001) and no significant relationship for males (*r* = 0.202, *P* = 0.437) (Fig. 6).

## Discussion

In this study, we investigated natural infection dynamics of two *Wolbachia* strains in *Aedes albopictus* mosquitoes collected across two Hawaiian Islands. These populations are characterized by high rates of *w*AlbB infection (> 98%), and only moderate levels of *w*AlbA (28.6%). We highlight that different factors shape the prevalence of each of these *Wolbachia* strains. For *w*AlbA, high prevalence is associated with sites experiencing higher temperatures and precipitation. However, *w*AlbB prevalence is driven by island and by precipitation, but not by sex or temperature. We also demonstrate that the density, or titer, of *w*AlbB is higher than that of *w*AlbA, higher in females than in males, and higher in co-occurrence with *w*AlbA. Together, these results reveal a highly asymmetric and heterogeneous *Wolbachia* infection structure, with important implications for both the ecological dynamics of co-infecting strains and the design of *Wolbachia*-based control strategies.

The previous study showed that 9 females collected from the Island of Hawai‘i were all positive for both *w*AlbA and *w*AlbB [8], which has an insufficient sample size to represent the infection dynamics of a population. To address the limitations in sample size and geographic range of the previous study, we expanded our sampling across multiple sites on O‘ahu and the Island of Hawai‘i, incorporating a substantially larger number of samples. Even with this expanded sampling effort, we observed a similarly high *Wolbachia* prevalence, with 98.5% of tested individuals being positive (N = 399/406). Breaking this down to consider only those specimens collected on the Island of Hawai‘i, we observed a *w*AlbB infection rate of 94.9% (37/39) and a *w*AlbA infection rate of 38.5% (15/39), with the latter contrasting to the 100% rate of superinfection observed in the previous study [8]. This disparity in wAlbB may reflect either a lower sample size of the earlier study, as raised in a previous study [29], or genuine temporal changes in infection dynamics. It is also possible that these observations reflect a real decline in *w*AlbA infection in the location over the past two decades.

Due to dramatic elevation gradients on the Island of Hawai‘i, there are diverse climatic conditions, creating nearly all climate zones [42]. Such climatic variation can contribute to differences in *Wolbachia* infection patterns across different sampling sites [43]. Our results revealed a strain-specific association between climatic variables and *Wolbachia* infection dynamics. Precipitation was the consistent climatic predictor, showing positive associations with *w*AlbA prevalence, *w*AlbB prevalence, and *w*AlbB density. Our study is one of the few studies to show the influence of precipitation on *Wolbachia* infection dynamics in mosquitoes. Previous studies on *Drosophila melanogaster* have shown context-dependent positive or negative associations, suggesting that the impact of precipitation on *Wolbachia* may vary across environments, or perhaps due to *Wolbachia* strains [44]. While temperature has been recognized as a key environmental factor shaping *Wolbachia* infection patterns [43, 45, 46], it was only associated with *w*AlbA prevalence in our study, suggesting that its influence may also be strain specific. These interactions between climatic variables and *Wolbachia* strains merit further investigation covering broader regions to see if this is unique to Hawai‘i or broadly applicable to other geographic regions.

*Wolbachia* infection patterns can shift over time due to environmental interactions and differential strain fitness. For instance, *Drosophila simulans* populations from the Australian east coast have been surveyed for *Wolbachia* infection of two strains, *w*Ri and *w*Au, over a period of about 20 years [47]. In the 1994 survey, *w*Au was common and *w*Ri absent, but a second survey in 2011–2012 showed that *w*Au infection rates had greatly declined while *w*Ri proliferated in its place. Temporal shifts in *Wolbachia* infection have also been documented in *D. melanogaster*, where the *w*Mel strain has replaced previously established *Wolbachia* strains across worldwide populations over several decades [48]. Therefore, it is plausible that natural *Wolbachia* infection patterns in Hawai‘i may also have changed over time. Notably, all *Wolbachia*-positive individuals carried *w*AlbB, while there were no cases of single infection with *w*AlbA, indicating a strong asymmetry in strain infection. The dominance of *w*AlbB over *w*AlbA observed in this study has been reported in other studies of *Ae. albopictus* across a broad geographic range [30, 31, 45, 49–52], suggesting that *w*AlbB dominance represents a common infection dynamic between the strains rather than a Hawaiian population-specific anomaly. Taken together, these findings point to a consistent asymmetry between strains, with *w*AlbB acting as the dominant and more broadly distributed infection, while *w*AlbA appears more restricted. While we provide the snapshot of *Wolbachia* strain in *Ae. albopictus*, it remains to be seen if this pattern is stable over the next few decades in the same location.

Although *w*AlbA and *w*AlbB naturally co-occur in *Ae. albopictus*, their prevalence was independent, whereas their relative densities were positively correlated, particularly in females. This infection pattern may be explained by the tissue-specific localization of *w*AlbA, which exhibits strong ovarian tropism, in contrast to *w*AlbB, which is distributed across a broader range of tissues, including both reproductive and somatic tissues [49, 53, 54]. Consistent with this explanation, *Wolbachia* infection is strongly sex-biased in this study, with *w*AlbA observed at higher prevalence and females showing higher density and superinfection rates than males. However, as this study used whole-body samples, tissue tropism was not directly examined, and therefore this interpretation should be made with caution. Further study on the tissue tropism may illuminate the mechanisms underlying the sex-biased infection patterns observed in *Ae. albopictus* populations in Hawai‘i.

The positive correlation between *w*AlbA and *w*AlbB is a plausible outcome given that long-term co-association of microorganisms is expected to be more mutualistic, as strong resource competition would likely prevent the persistence of stable superinfections. Positive co-associations between strains might reflect a shared response to host or environmental conditions promoting high prevalence and density of *Wolbachia* in *Ae. albopictus* [10, 45, 49, 55]. Under such environmental conditions, both *w*AlbA and *w*AlbB may reach high density. This positive co-association between *w*AlbA and *w*AlbB density is consistent with observations from *Ae. albopictus* populations in the Indo-Pacific region [30], supporting the idea that strain co-occurrence without direct competition is a general feature across *Ae. albopictus* populations. Such stable co-occurrence suggests that strong competitive exclusion between strains is limited, potentially reflecting long-term co-adaptation within the host. Nonetheless, the consistent dominance of *w*AlbB indicates that competitive hierarchies may still exist at the within-host level, and the asymmetric relative proportions of *w*AlbA and *w*AlbB may also reflect on the relative competitiveness within a mosquito, and it is plausible that we may see a different balance of *w*AlbA and *w*AlbB decades later. Long-term monitoring of the *Wolbachia* infection status may illuminate the competitiveness of different *Wolbachia* strains in the future.

The high level of genetic similarity observed among *Ae. albopictus* populations in the Hawaiian Islands may be one of the major factors contributing to the consistent *Wolbachia* infection patterns across multiple islands. Population genomic analysis revealed that *Ae. albopictus* populations are genetically similar throughout the Hawaiian Islands, including O‘ahu and the Island of Hawai‘i, despite geographical isolation by the surrounding ocean [55]. Given the high genetic similarity among *Ae. albopictus* populations in Hawai‘i and their consistent *Wolbachia* infection patterns, it is plausible that similar *Wolbachia* infection patterns occur on other Hawaiian Islands, such as Maui and Kauai, although further investigation is needed to confirm.

The overall infection pattern we observed across the two islands, 28.6% *w*AlbA-infected and 98.5% *w*AlbB-infected, has important implications for the design of IIT strategies utilizing CI. This heterogeneous infection structure creates a complex compatibility landscape that may limit the effectiveness of IIT strategies relying on single-strain releases. If mosquitoes in the region are a mix of superinfected, *w*AlbB-infected, and uninfected, for any IIT intervention to succeed, a release line must induce CI against female mosquitoes with any of these infection statuses. Bidirectional incompatibility in these cases is complex, as evidenced by the fact that mating between *w*AlbA- and *w*AlbB- mono-infected *Ae. albopictus* can also induce CI [57]. Several studies have explored alternative *Wolbachia* transinfections for this purpose, including *w*Mel [58], and *w*Pip [59] after first clearing the two native strains. Meanwhile, an additional *Wolbachia* strain can be overlayed on the two native strains to create a triple infection that induces incompatibility with the native strains [36]. Host and strain genetics, and insecticide resistance status are also important considerations in any intervention as they can impact post-release host performance, and ultimately, the success of an intervention [60, 61].

Our earlier studies highlight the fact that *Ae. albopictus* populations in Hawai‘i have high genetic similarity [56] and exhibit no strong genotypic or phenotypic evidence of permethrin resistance [62]. Consequently, if a single *Wolbachia*-transinfected *Ae. albopictus* line were to be produced in a Hawaiian genetic background, it could potentially be used broadly across the Hawaiian Islands. However, if *Ae. albopictus* populations in Hawai‘i share a common genetic source, island effect models are more supportive of the genetic divergence of *Wolbachia* from the ancestral population, rather than within the population [63]. As such, there is a need to characterize the genetic similarity amongst the two *Wolbachia* strains prevalent in Hawai‘i, particularly between islands.

## Conclusions

This study investigated natural *Wolbachia* infection patterns in the *Aedes albopictus* populations of two Hawaiian Islands, demonstrating that the population is of mixed infection status with a moderate *w*AlbA prevalence and high *w*AlbB prevalence. Interestingly, levels of *w*AlbA contrast with the expectation based on the previous study in Hawai‘i. We also demonstrate sex-specific differences in superinfection rates, driven by low *w*AlbA infection rates amongst males. Our results indicate that *Wolbachia* prevalence and density vary with climatic conditions in a strain-specific manner, highlighting the potential role of environmental heterogeneity in shaping *Wolbachia* infection patterns in this important mosquito vector. These dynamics highlight a need for characterization of mating incompatibility in advance of future *Wolbachia*-mediated population suppression interventions in the region.

## Supplementary Material

Supplementary Files

This is a list of supplementary files associated with this preprint. Click to download.

• TableS1.PresenceanddensityofWolbachiastrainsinAedesalbopictusinHawaii.xlsx

## Figures and Tables

**Table 1:** Distribution of *Wolbachia*-infected *Aedes albopictus* by island, sampling site, and sex.

Island	Sites	Latitude	Altitude	Sex	No. of sample	No. of infected individual		
						Uninfected	*w*AlbB only	Superinfected
O‘ahu	Aliamanu Military Reservation (AMR)	21.359	−157.922	F	9	0 (0)	3 (33.3)	6 (66.7)
				M	8	0 (0)	8 (100)	0 (0)
	Fort Shafter (FS)	21.339	−157.888	F	38	0 (0)	8 (21.1)	30 (78.9)
				M	24	0 (0)	21 (87.5)	3 (12.5)
	Helemano (HE)*	21.534	−158.021	F	3	0 (0)	3 (100)	0 (0)
				M	4	0 (0)	4 (100)	0 (0)
	Mānoa (MA)*	21.305	−157.818	F	10	0 (0)	10 (100)	0 (0)
				M	2	0 (0)	2 (100)	0 (0)
	Mililani Mauka (MM)	21.488	−158.014	F	21	1 (4.8)	8 (38.1)	12 (57.1)
				M	28	0 (0)	25 (89.3)	3 (10.7)
	Schofield Barracks (SB)	21.493	−158.060	F	14	0 (0)	7 (50.0)	7 (50.0)
				M	12	0 (0)	11 (91.7)	1 (8.3)
	Tripler Army Medical Center (TAMC)	21.360	−157.889	F	82	0 (0)	54 (65.9)	28 (34.1)
				M	73	1 (1.4)	63 (86.3)	9 (12.3)
	Wheeler Army Airfield (WA)*	21.485	−158.039	F	4	0 (0)	3 (75.0)	1 (25.0)
				M	3	0 (0)	3 (100)	0 (0)
	Total			F	181	1 (0.6)	96 (53.0)	84 (46.4)
				M	154	1 (0.6)	137 (89.0)	16 (10.4)
Island of Hawai‘i	Hilo (HI)*	19.700	−155.085	F	1	0 (0)	1 (100)	0 (0)
				M	1	0 (0)	1 (100)	0 (0)
	Hōlualoa (HO)	19.595	−155.947	F	12	0 (0)	10 (83.3)	2 (16.7)
				M	5	0 (0)	5 (100)	0 (0)
	Miloli’i (MI)	19.188	−155.905	F	21	1 (4.8)	9 (42.9)	11 (52.4)
				M	12	0 (0)	12 (100)	0 (0)
	Kawaihae (KA)	20.037	−155.826	F	5	1 (20.0)	2 (40.0)	2 (40.0)
				M	13	2 (15.4)	10 (76.9)	1 (7.7)
	Total			F	39	2 (5.1)	22 (56.4)	15 (38.5)
				M	31	2 (6.5)	28 (90.3)	1 (3.2)
Total					405	6 (1.5)	283 (69.9)	116 (28.6)

Infection rates (%) are indicated in parentheses. Asterisk indicates populations were excluded from the statistical analysis due to the small number of samples.

## Data Availability

All data supporting the findings of this study are available within the paper and its Supplemental Table S1.
